# Prevalence and Associated Factors of Psychological Distress and Burnout among Medical Students: Findings from Two Campuses

**DOI:** 10.3390/ijerph18168446

**Published:** 2021-08-10

**Authors:** Nik Muhammad Nik Ahmad Arif, Nurhanis Syazni Roslan, Shaiful Bahari Ismail, Ramyashilpa D. Nayak, Muhamad Ridzuan Jamian, Alya Syahmina Mohamad Ali Roshidi, Teh Chen Edward, Muhammad Aiman Kamal, Muhammad Mujaahid Mohd Amin, Shukri Shaari, Muhammad Fikri Shaharudin Basri

**Affiliations:** 1 School of Medical Sciences, Universiti Sains Malaysia, Kota Bharu 16150, Kelantan, Malaysia; nmarif.umed15@student.usm.my (N.M.N.A.A.); shaifulb@usm.my (S.B.I.); syahminaali@gmail.com (A.S.M.A.R.); edward6860@icloud.com (T.C.E.); aimankamal@usm.my (M.A.K.); mujaahidamin@gmail.com (M.M.M.A.); shukrishaari96@gmail.com (S.S.); mfikri94@yahoo.com (M.F.S.B.); 2 USM-KLE International Medical Program Campus, Belgaum 590010, Karnataka, India; ramyanarahari@usm.my (R.D.N.); ridzuanjamian96@gmail.com (M.R.J.)

**Keywords:** burnout, psychological distress, medical student, prevalence, student well-being

## Abstract

Medical training is intensive and predisposes students to psychological distress and burnout. Unaddressed burnout in medical training may persist in the internship phase and impact the quality of patient care. While some associations have been established, the link between some individual factors and training characteristics with distress and burnout in medical training remained unclear. In this study, we aim to examine the prevalence of psychological distress and burnout, and its association with gender, training phase, funding status, cumulative grade points average (CGPA), and coping strategies among medical students. The study applied a multicenter cross-sectional study design and convenience sampling on medical students from two medical schools from Malaysia and India. We used a self-reporting instrument that includes demographic details, the 12-item General Health Questionnaire (GHQ), the Copenhagen Burnout Inventory (CBI), and the Brief Coping Orientation to Problems Experienced (Brief COPE). A total of 748 medical students participated in the study. The prevalence of psychological distress, personal-related, work-related, and patient-related burnout were 33.0%, 56.1%, 35.0%, and 26.2%, respectively. Being male, clinical year, self-funded, and having a CGPA of more than 3.50 predicted psychological distress and burnout with mixed results. Maladaptive coping mechanisms consistently predicted the risk of psychological distress and burnout by more than two times. The findings indicate that primary and secondary mental health interventions have a role in medical training. A systematic intervention should incorporate coping skills training alongside institutional-targeted intervention.

## 1. Introduction

Medical school curricula are designed to equip students with the knowledge, skills, and professionalism required to produce competent doctors. Unfortunately, the intensive nature of medical training may jeopardize student’s mental health. Studies have shown that psychological distress in medical students is higher when compared to the general population [1,2,3,4,5,6]. Previous literature proposed that the practice and environment of the training contribute to an array of stressors, which can lead to high psychological morbidity [1,2,7]. This is evident by a meta-analysis that found a 13.5% increase in depression symptoms among medical students after entering medical schools [8]. This is a worrying trend as higher rates of distress are linked to poor academic performance, increased dropout, decreased empathy, and suicidal ideation [9,10,11,12].

To make matters worse, unaddressed mental health problems may lead to burnout among medical students [13]. Burnout syndrome resulted from a chronic workplace or academic stress and is characterized by exhaustion that is followed by depersonalization and cynical attitude and reduced personal accomplishment [14]. For the past decade, burnout is increasingly studied and the prevalence of burnout in medical and surgical residents had been reported up to 80% [15,16,17].

Studies have examined the associations between several demographic factors and mental health problems in medical training. While some studies found female students had higher psychological distress [18], a meta-analysis found no significant correlation between gender and burnout [19]. Uncertainties for the future, educational constraints, and personal problems were linked to a higher risk of psychological distress [20]. Younger age was consistently associated with a higher risk of burnout among physicians [21,22]. A review conducted in 2013 proposed different stressors across medical training. Burnout among medical students in preclinical years was associated with the level of support from faculty staff, while burnout among clinical-year students was associated with clerkship and cynical residents [23]. This raised the question of whether stress and burnout are more prevalent in certain stages or persists throughout the training. Emotional intelligence has been consistently associated with lower burnout [24,25]. However, less is known whether cognitive abilities that are highly sought after in medical students protect them from psychological distress and burnout.

On top of these demographic factors, coping strategies have been linked to psychological distress and burnout through several central theories. The transactional theory of stress and coping defined coping as cognitive and behavioral efforts to address, tolerate, or reduce external or internal demands, and the conflicts that arise from them. Coping plays an important role in the appraisal process of individuals and may contribute to the variability of responses among individuals facing similar sources of stress [26]. The coping reservoir model posits that each individual has a coping reservoir that can deplete or fill repeatedly, and the inability to replenish the reservoir could contribute to psychological distress and burnout. In medical training, it is important to establish whether certain training characteristics act as replenishing or depleting factors to students’ reservoir when facing challenges [13].

The impact of psychological distress and burnout could influence medical students’ practice habits. Unaddressed burnout may persist and will not just affect the students’ quality of life but also their competency as healthcare professionals in the future [27,28]. Building on the above conceptual framework, we set out to investigate the prevalence of psychological distress and burnout, and its association with individual factors and training characteristics among medical students. We hypothesized that the prevalence of psychological distress and burnout is high among medical students, and it is significantly higher among female students, preclinical-year students, self-funded students, students with lower cumulative grade points average (CGPA), and students practicing higher maladaptive coping strategies. These understandings may inform medical schools on the relevant aspects to be addressed during the training and suitable prevention measures.

## 2. Materials and Methods

### 2.1. Study Design

The study utilized a multicenter, cross-sectional design, and data collection was conducted from January to April 2018.

### 2.2. Participants

The sampling frame involved 686 medical students from Universiti Sains Malaysia (USM) Health Campus Kubang Kerian, Kelantan, Malaysia, and 472 medical students from Universiti Sains Malaysia–Karnataka Lingayat Education (USM-KLE) International Medical Program, Belgaum, India. Although located in different geographical locations, these two campuses shared the same curriculum structure. The medical training was divided into preclinical (year 1 and 2) and clinical phases (year 3 to 5). Most of the students from the two campuses were Malaysians. Ethical approval was obtained from both schools (USM/JEPEM/1711066) prior to the data collection.

The required sample size for a population of 1158, with a confidence level of 99%, a margin of error of 0.05, and the non-response rate of 50% was 846. Our participants were drawn from a convenience sample within the population. Data collection was performed outside the revision week to prevent the possible effects of confounding factors such as examination pressure. We approached the leader of each group in each year to set a schedule for distributing the questionnaire. Eligible students were invited to participate in the studies after finishing their lectures or clinical teaching.

Participation was voluntary and anonymous. Informed consent was obtained, and data collection was performed by paper and pen, which took an average of 20 min to fill in. The session was managed by one of the researcher team members who had no authority or supervisory role on the students. Participants were given the opportunity to know their own psychological distress and burnout scores as an incentive. Students that did not give their consent were excluded from the study.

### 2.3. Study Instruments

The questionnaire included demographic details such as campus, gender, study year, study funding, and participant’s CGPA. It also included the 12-item General Health Questionnaire (GHQ), the Copenhagen Burnout Inventory (CBI), and the Brief Coping Orientation to Problems Experienced (Brief COPE). The questionnaire package was in English, as it is the language used in the medical curriculum.

The 12-item GHQ was selected as it is a well-established unidimensional measure of psychological distress and is widely used in general and clinical populations worldwide. GHQ-12 comprises 12 items describing mood states, six of which are positively phrased and six are negatively phrased [29]. Responses were assessed using the Likert method in which the maximum score is 36 with each item being in the range of zero (not at all) to three (much more than usual). The higher scores indicate an increase in distress, which leads to a decrease in quality of life [30]. Participants scoring a mean of more than 4 are considered as “psychiatric caseness” [31]. The GHQ-12 has excellent reliability ranging from 0.78 to 0.95 and has been validated in the studied population [30].

The CBI is a freely accessible tool to measure burnout in three domains that are personal-related (six items), work-related (seven items), and patient-related burnout (six items) [32]. In contrast to the Maslach Burnout Inventory that measures exhaustion as part of its domain, the CBI regards exhaustion as the central component of burnout [33]. Each item is rated on a Likert scale that ranges from “always” (100 points) to “never” (0 points) (for personal- and work-related domains) or “a very high degree” (100 points) to “a very low degree” (0 points) (for patient-related domain). We treated the continuous scores dichotomously, in which the mean score of 50 and above for each domain are considered burnout [34]. Based on previous studies, CBI has very high reliability and has been validated in the studied population [32,35].

The COPE inventory, developed by Carver, Scheier, and Wentraub (1989) is a comprehensive 60 items questionnaire that measures 15 coping styles and strategies. Carver later developed the Brief COPE, a more concise version to reduce the time taken by the participants to answer the inventory. The questionnaire comprises a 28-items inventory that measures 14 coping styles and strategies. Participants were asked to score each item on a Likert scale of “not doing it at all” (1) to “doing it a lot” (4) [36]. We divided the items into 3 subscales, which are problem-focused coping (active coping, planning, and use of instrumental support), emotion-focused coping (use of emotional support, positive reframing, acceptance, religion, and humor), and maladaptive coping (venting, denial, substance use, behavioral disengagement, self-distraction, and self-blame) [37]. Participants’ scores for each subscale were summed and averaged.

### 2.4. Statistical Analysis

All statistical analyses were performed using Software for the Social Sciences (SPSS) version 27 (IBM Corp., Armonk, NY, USA). Any missing values were replaced by the median of all the values in the items. For the primary analysis, we used descriptive statistics to estimate the prevalence of psychological distress and burnout among the participants. Next, we examined the association of demographic factors with psychological distress and burnout by using simple logistic regression. Only variables that showed a *p*-value of 0.20 or less were included in the final model of multivariable regression [38]. For multivariable analysis, binary logistic regression was performed to predict the effect of several independent variables on mental health problems and burnout. We detected no collinearity for the independent variables. We also reported the receiver operating characteristic (ROC) curve to assess the accuracy of the model prediction. Based on Mandrekar, 2010 [39], area under the ROC curve (AUC) value of 0.7 to 0.8 is considered acceptable, and 0.8 to 0.9 is regarded as excellent, while 0.9 and above is outstanding.

## 3. Results

### 3.1. Demographic Distribution

Based on the demographic distribution in Table 1, a total of 748 (67.7%) out of 1158 medical students from both universities completed the inventories. Of those participating in the study, 500 students (72.9%) were from USM Malaysia, and 248 students (52.5%) were from USM-KLE. Participants were more likely to be female (67.5%), which followed the national report on human resources in Malaysia, according to which 61.2% of all doctors in Malaysia were female [40]. In total, 45.9% of the participants were in their preclinical years, and the rest of the participants (54.1%) were clinical-phase students. In addition, 78.1% of the participants received scholarships during their studies. Regarding CGPA, 12.7% of the participants achieved a cumulative grade point average (CGPA) of more than 3.5, and 54.7% obtained a CGPA of less than 3.49, while 32.6% did not have a CGPA yet (first-year students) or did not report their CGPA.

### 3.2. Prevalence of GHQ Caseness in Medical Students

As shown in Table 2, the overall prevalence of GHQ caseness (GHQ ≥ 4) for medical students was 33%. USM Malaysia had a higher caseness when compared to USM-KLE (39.6% and 19.8%, respectively). Medical students from the first year had the highest prevalence (38.1%) among all the batches, followed by the fifth year (37.4%) and third-year students (34.6%). Nearly the same prevalence of caseness was reported for preclinical and clinical year students (32.1% and 33.8%, respectively), and male and female students (34.6% and 32.3%, respectively). A lower prevalence was observed in students receiving scholarships when compared to self-funding medical students (29.3% and 46.3%, respectively). A higher rate of caseness was observed in students with a CGPA between 3.50 and 4.00 (35.8%), as compared to students with a CGPA of less than 3.49 (28.6%).

### 3.3. Prevalence of Burnout in Medical Students

As shown in Table 2, the overall prevalence for personal-, work-, and patient-related burnout were 56.1%, 35.0%, and 26.2%, respectively. Comparing between two campuses, USM Malaysia had a higher prevalence of personal- and work-related burnout. USM-KLE had a higher patient-related burnout. Third-year students had the highest personal-related burnout (66.0%), while fifth-year students had the highest prevalence for both work-related and patient-related burnouts (46.0% and 28.1%, respectively). Clinical-year students had a higher personal-related burnout (64.0% vs. 46.9%) and work-related burnout (43.2% vs. 25.4%) when compared to those in the preclinical phase. However, with regard to patient-related burnout, the prevalence was the same for both phases. Female students had a higher rate of personal-related burnout (57.4% vs. 53.5%), while male students had a higher prevalence for work-related (42.4% vs. 31.5%) and patient-related burnout (37.0% vs. 21.0%). When compared to the self-paying students, students receiving scholarships had a higher personal-related burnout (56.3% vs. 55.5%) but a lower work-related (34.2% vs. 37.8%) and patient-related burnout (26.0% vs. 26.8%). High-achieving students with a CGPA of more than 3.50 to 4.00 had a higher prevalence in personal-related (57.9% vs. 57.2%), work-related (44.2% vs. 34.5%), and patient-related burnout (29.5% vs. 26.9%) when compared to students with CGPA < 3.49.

### 3.4. Relationship between Demographic, Coping Strategies, and Psychological Distress

As illustrated in Table 3, self-funding students were 2.09 times (95% CI: 1.46, 2.98) more likely to experience psychological distress during their studies, as compared to students who received scholarships. With a one-point increase in problem-focused and emotion-focused coping scores, the odds ratio of psychological distress decreased 24% and 26%, respectively. However, with a one-point increase in maladaptive coping, students were 2.77 times (95% CI: 2.20, 3.50) more likely to experience psychological distress.

### 3.5. Relationship between Demographic, Coping Strategies, and Burnout

Male students were more likely to experience work- and patient-related burnout (odds ratio (OR): 1.60, 95% CI: (1.17, 2.20)) and (OR: 2.21, CI 95%: (1.58, 3.10)), respectively. Students in the clinical phase were twice more likely of having personal-related burnout (OR: 2.01, 95% CI: (1.50, 2.69)) and work-related burnout (OR: 2.24, 95% CI: (1.64, 3.06)). No significant relationship was found for scholarships status or CGPA. Focusing on the coping mechanism of the medical student, a one-point increase in problem-focused coping lowered the risk of all personal-related, work-related, and patient-related burnout by 21%, 32%, and 24%, respectively. A one-point increase in emotion-focused coping lowered the risk of having personal-related, work-related, and patient-related burnout by 22%, 28%, and 16%. One-point increase in maladaptive coping increased the likelihood of personal-related, work-related, and patient-related burnout (OR: 2.33, 95% CI: (1.87, 2.92)), (OR: 2.12, CI 95%: (1.71, 2.62)), and (OR: 1.94, CI 95%: (1.56, 2.40)), respectively.

### 3.6. Multivariable Analysis of Psychological Distress and Burnout

Significant multivariable relationships are shown in Table 3. Self-funding students (adjusted OR (aOR) 2.03, 95% CI: (1.37, 3.00)) and maladaptive coping (aOR 3.29, 95% CI: (2.54, 4.27)) were positively associated with psychological distress while emotion-focused coping (aOR 0.63, 95% CI: (0.51, 0.79)) was negatively associated with psychological distress. Clinical-year students (aOR 2.16, 95% CI: (1.57, 2.99)) and maladaptive coping (aOR 2.89, 95% CI: (2.25, 3.72)) were positively associated with personal-related burnout, while emotion-focused coping (aOR 0.71, 95% CI: (0.58, 0.87)) was negatively associated with personal-related burnout. Male gender (aOR 1.63, 95% CI: (1.07, 2.48)), clinical-year students (aOR 2.32, 95% CI: (1.48, 3.63)), and maladaptive coping (aOR 2.54, 95% CI: (1.90, 3.40)) were positively associated with work-related burnout, while emotion-focused coping (aOR 0.75, 95% CI: (0.58, 0.97)) were negatively associated with work-related burnout. Lastly, male gender (aOR 2.21, 95% CI: (1.55, 3.14)) and maladaptive coping (aOR 2.04, 95% CI: (1.62, 2.57)) were positively associated with patient-related burnout while problem-focused coping (aOR 0.79, 95% CI: (0.65, 0.97)) was negatively associated with patient-related burnout. The AUC value for psychological distress, personal-related, work-related, and patient-related burnout multivariable models were acceptable (0.738, 0.716, 0.745, and 0.702, respectively).

## 4. Discussion

USM currently offers a program of Medical Doctor (MD) held in two campuses: USM Health Campus in Malaysia and USM-KLE in India. Although both of the campuses have the same curriculum and exams in terms of preclinical and clinical work, some education environment factors such as facilities, amount of scholarship funds received, and also different cultures surrounding the campuses could have contributed to the difference in the prevalence. This cross-sectional study accords to a number of studies [10,11,41] that reported a higher response rate from early study years of medical school. This is probably due to the nature of the preclinical-phase lectures, which are attended by the whole batch (thus ensuring a higher response rate), as compared to the clinical phase during which students attend teaching in a smaller group.

The current study reported a psychiatric caseness of 33%, which was lower than the prevalence obtained from a sample of medical students in a national study (50%) [42]. However, this figure is higher than the prevalence of caseness among adolescents (25%) [43]. With regard to burnout, a systematic review reported medical students’ overall burnout prevalence of 45% to 71% [23]. A meta-analysis published in 2019 on 35 studies reported work-related burnout prevalence among medical students ranged from 7.0% to 75.2% [44]. The huge range of prevalence was due to the different instruments used in measuring the rate of burnout and different cut-off criteria in determining burnout among medical students. When compared to the systematic review by Erschens et al. (2019), the present study reported a lower work-related burnout prevalence (35%) than other studies that used CBI (41% to 51%). While there were limited studies that compared medical students and the general population using CBI, we found that the prevalence obtained from this sample was higher than non-medical students (20.1%) [45]. However, the prevalence of personal-related, work-related, and patient-related burnout was declining when compared to a study conducted among USM medical students of previous curriculum structure. Personal-related burnout remained as the domain with the highest prevalence, followed by work-related, and patient-related burnout [46].

Contrary to our hypothesis, male medical students were more likely to experience work-related and patient-related burnout, as compared to female students. Other studies reported no significant difference between gender and burnout [46,47], and a study using CBI showed a mixed result in which females scored significantly higher than men in personal- and work-related burnout [34]. While the reason behind this is unclear, explanation from the literature proposed that male students may struggle in a female-typed occupation such as doctors and other caregiving professions, which are filled with emotional and interpersonal task demands [48].

In contrast to studies that shown a higher burnout among younger physicians [21,22], we found that clinical phase students were twice more likely to experience personal and work-related burnout, as compared to their junior colleagues in the preclinical phase. This could be explained by the higher workload in their daily schedules as they need to join ward rounds, complete their posting requirements, and have to attend night calls in addition to their classes on the next day, which could lead to physical and mental exhaustion. Clinical phase students also have less time for themselves in order to cope with their personal problems and that could result in higher personal-related burnout. Similar findings were reported by studies conducted in Asian and Western contexts [46,49].

In terms of academic performance and burnout, hypothetically higher burnout is associated with underachieving students [50]. Contrary to expectation, we found that students with a CGPA of more than 3.50 were at higher risk for work-related burnout, as compared to the lower-achieving students. Striving to achieve an excellent result could have led students to physical and emotional exhaustion. A similar phenomenon was described in the earliest study of burnout, which indicated that it affected the most dedicated workers who committed more intensely in their undertaking [51]. If this is left unchecked, affected students may start to develop cynical attitudes toward their studies, lecturers, and also their clinical work [50].

It is reassuring to find that with good coping mechanisms, the risk of mental health problems and burnout could be reduced up to 40% in medical students. The risk reduction in this study for both problem- and emotion-focused coping were consistent, even though there was a slight variation of odds ratio between the two strategies. This is in accordance with studies that reported the importance of the emotion-focused role in the coping strategy [52,53]. However, prolonged use of high emotion-focused coping without active coping strategies would eventually lead to emotional fatigue [54]. Developing coping strategies such as direct problem-focused in accordance with emotion-focused would help students to become more effective in coping with the sources of psychological distress [55]. A study of colorectal and vascular surgeon coping mechanisms found that having less time with family and friends, and keeping problems to themselves increased the risk of stress and burnout levels [52,56]. Maladaptive coping mechanisms were more likely to increase mental health problems and burnout. Students who utilize maladaptive coping usually would not deal with their problems in a constructive manner. Although maladaptive coping may temporarily suppress stressful situations and distract students from the sources of stress, it could eventually lead to emotional exhaustion through loss spirals [56,57].

Our findings could also be explained by the coping reservoir model, which outlines the importance of coping for the well-being of medical students [13]. Each student has a coping reservoir that is continuously filled and drained as they faced challenges. Negative input (e.g., stress, internal conflicts, and time and energy demands) depletes the reservoir and positive input (e.g., psychosocial support, social activities, mentorship, and intellectual stimulation) can replenish the reservoir. Throughout the studying years, the coping reservoir will be drained and filled repeatedly, and the failure to replenish the reservoir through coping or other mechanisms may cause them to experience negative outcomes (burnout, frustration, and cynicism) [13].

### Limitations of Study

Strengths of this study include its multicenter study sites, large sample size, and the use of validated instruments. As with other studies, this study also had a few limitations. Firstly, this study used non-probability sampling, which limits its generalizability to the population. This study also did not capture institutional factors such as the learning environment, teaching methods used by the lecturers, and supervision quality. On top of that, the findings from the cross-sectional study were not causal effects. Lastly, there might be a possibility of reporting bias from the use of self-reporting instruments. The study was conducted before the COVID-19 pandemic. Hence, another study is desirable to estimate the prevalence of psychological distress and burnout and significant associations in this novel situation. Despite the limitations, these findings offer insight into psychological distress and burnout among medical students and some guidance for possible interventions.

## 5. Conclusions

This study highlights that 26.2% to 56.1% of medical students in USM Malaysia and USM-KLE campuses experienced psychological distress or burnout. Being male, clinical year, self-funded, and having a CGPA of more than 3.50 significantly predicted psychological distress and burnout with mixed results. However, maladaptive coping mechanisms consistently predicted the risk of developing mental health problems and burnout by more than two times. Therefore, primary and secondary interventions should be studied and integrated with the medical curriculum, as unaddressed burnout was found to impact individuals for one to three years [24,58]. Unaddressed burnout may also persist in internships (burnout hangover) and may impact the quality of patient care [28]. Hence, proactive measures must also be taken to impart coping skills alongside other interventions.

## Figures and Tables

**Table 1:** Study participants demographic distribution.

Demographic Variable (*n* = 748)	% (*n*)
Campus	
USM Malaysia	66.8 (500)
USM-KLE	33.2 (248)
Year	
1	25.3 (189)
2	20.6 (154)
3	21.7 (162)
4	13.9 (104)
5	18.6 (139)
Phase	
Preclinical	45.9 (343)
Clinical	54.1 (405)
Gender	
Male	32.5 (243)
Female	67.5 (505)
Funding	
Scholarship	78.1 (584)
Self-funding	21.9 (164)
CGPA	
3.50–4.00	12.7 (95)
<3.49	54.7 (409)
No CGPA yet/missing data	32.6 (244)

**Table 2:** Mental health problems and burnout prevalence for overall and each demographic variable (*n* = 748).

Variables	GHQ Caseness ≥ 4	Personal-Related Burnout	Work-Related Burnout	Patient-Related Burnout
	% (*n*)	% (*n*)	% (*n*)	% (*n*)
Overall	33.0 (247)	56.1 (420)	35.0 (262)	26.2 (196)
Campus				
Malaysia	39.6 (198)	58.4 (292)	38.4 (192)	25.6 (128)
India	19.8 (49)	51.6 (128)	28.2 (70)	27.4 (68)
Year				
1	38.1 (72)	52.4 (99)	29.1 (55)	26.5 (50)
2	24.7 (38)	40.3 (62)	20.8 (32)	26.0 (40)
3	34.6 (56)	66.0 (107)	42.6 (69)	24.7 (40)
4	27.9 (29)	62.5 (65)	40.4 (42)	26.0 (27)
5	37.4 (52)	62.6 (87)	46.0 (64)	28.1 (39)
Phase				
Preclinical	32.1 (110)	46.9 (161)	25.4 (87)	26.2 (90)
Clinical	33.8 (137)	64.0 (259)	43.2 (175)	26.2 (106)
Gender				
Male	34.6 (84)	53.5 (130)	42.4 (103)	37.0 (90)
Female	32.3 (163)	57.4 (290)	31.5 (159)	21.0 (106)
Funding				
Scholarship	29.3 (171)	56.3 (329)	34.2 (200)	26.0 (152)
Self-funding	46.3 (76)	55.5 (91)	37.8 (62)	26.8 (44)
CGPA				
3.50–4.00	35.8 (34)	57.9 (55)	44.2 (42)	29.5 (28)
<3.49	28.6 (117)	57.2 (234)	34.5 (141)	26.9 (110)

**Table 3:** Univariate and multivariable logistic regression of relevant demographic variables and coping strategies with mental health problems and burnout in medical students.

	GHQ Caseness		Personal–Related Burnout		Work–Related Burnout		Patient–Related Burnout	
Variables	UVA	MVA	UVA	MVA	UVA	MVA	UVA	MVA
	OR	aOR	OR	aOR	OR	aOR	OR	aOR
	(95% CI)	(95% CI)	(95% CI)	(95% CI)	(95% CI)	(95% CI)	(95% CI)	(95% CI)
Gender: Male					1.60 **	1.63 *	2.21 **	2.21 **
					(1.17–2.20)	(1.07–2.48)	(1.58–3.10)	(1.55–3.14)
Phase: Clinical			2.01 **	2.16 **	2.24 **	2.32 **		
			(1.50–2.69)	(1.57–2.99)	(1.64–3.06)	(1.48–3.63)		
Funding: Self	2.09 **	2.03 **						
	(1.46–2.98)	(1.37–3.00)						
CGPA:					1.51			
Above 3.50					(0.96–2.37)			
Coping strategies:								
Problem-focused	0.76 **		0.79 **		0.68 **		0.76 **	0.79 *
	(0.66–0.88)		(0.69–0.91)		(0.59–0.79)		(0.65–0.89)	(0.65–0.97)
Emotion-focused	0.74 **	0.63 **	0.78 **	0.71 **	0.72 **	0.75 *	0.84 *	
	(0.63–0.86)	(0.51–0.79)	(0.67–0.90)	(0.58–0.87)	(0.62–0.84)	(0.58–0.97)	(0.72–0.99)	
Maladaptive	2.77 **	3.29 **	2.33 **	2.89 **	2.12 **	2.54 **	1.94 **	2.04 **
	(2.20–3.50)	(2.54–4.27)	(1.87–2.92)	(2.25–3.72)	(1.71–2.62)	(1.90–3.40)	(1.56–2.40)	(1.62–2.57)

UVA: univariate analysis, MVA: multivariable analysis, OR: odds ratio, aOR: adjusted odds ratio, CI: confidence interval; *p*-value: no asterisk (0.20 < *p* < 0.05), * *p* < 0.05, ** *p* < 0.001.

## Data Availability

The data presented in this study are available on request from the corresponding author.

## References

[1] Dyrbye L.N., Thomas M.R., Huntington J.L., Lawson K.L., Novotny P.J., Sloan J.A., Shanafelt T.D. (2006). Personal life events and medical student burnout: A multicenter study. Acad. Med..

[2] Dyrbye L.N., Thomas M.R., Shanafelt T.D. (2006). Systematic review of depression, anxiety, and other indicators of psychological distress among US and Canadian medical students. Acad. Med..

[3] Hope V., Henderson M. (2014). Medical student depression, anxiety and distress outside North America: A systematic review. Med. Educ..

[4] Maser B., Danilewitz M., Guérin E., Findlay L., Frank E. (2019). Medical student psychological distress and mental illness relative to the general population: A Canadian cross-sectional survey. Acad. Med..

[5] Dahlin M., Joneborg N., Runeson B. (2005). Stress and depression among medical students: A cross-sectional study. Med. Educ..

[6] Wong J.G., Cheung E.P., Chan K.K., Ma K.K., Tang S.W. (2006). Web-based survey of depression, anxiety and stress in first-year tertiary education students in Hong Kong. Aust. N. Z. J. Psychiatry.

[7] Aktekin M., Karaman T., Senol Y.Y., Erdem S., Erengin H., Akaydin M. (2001). Anxiety, depression and stressful life events among medical students: A prospective study in Antalya, Turkey. Med. Educ..

[8] Rotenstein L.S., Ramos M.A., Torre M., Segal J.B., Peluso M.J., Guille C., Sen S., Mata D.A. (2016). Prevalence of depression, depressive symptoms, and suicidal ideation among medical students: A systematic review and meta-analysis. JAMA.

[9] Dyrbye L.N., Massie F.S., Eacker A., Harper W., Power D., Durning S.J., Thomas M.R., Moutier C., Satele D., Sloan J. (2010). Relationship between burnout and professional conduct and attitudes among US medical students. JAMA.

[10] Dyrbye L.N., Thomas M.R., Massie F.S., Power D.V., Eacker A., Harper W., Durning S., Moutier C., Szydlo D.W., Novotny P.J. (2008). Burnout and suicidal ideation among US medical students. Ann. Intern. Med..

[11] Thomas M.R., Dyrbye L.N., Huntington J.L., Lawson K.L., Novotny P.J., Sloan J.A., Shanafelt T.D. (2007). How do distress and well-being relate to medical student empathy? A multicenter study. J. Gen. Intern. Med..

[12] Stewart S.M., Lam T., Betson C., Wong C., Wong A. (1999). A prospective analysis of stress and academic performance in the first two years of medical school. Med. Educ..

[13] Dunn L.B., Iglewicz A., Moutier C. (2008). A conceptual model of medical student well-being: Promoting resilience and preventing burnout. Acad. Psychiatry.

[14] Maslach C., Schaufeli W.B., Leiter M.P. (2001). Job burnout. Annu. Rev. Psychol..

[15] Gopal R., Glasheen J.J., Miyoshi T.J., Prochazka A.V. (2005). Burnout and internal medicine resident work-hour restrictions. Arch. Intern. Med..

[16] Shanafelt T.D., Bradley K.A., Wipf J.E., Back A.L. (2002). Burnout and self-reported patient care in an internal medicine residency program. Ann. Intern. Med..

[17] Willcock S.M., Daly M.G., Tennant C.C., Allard B.J. (2004). Burnout and psychiatric morbidity in new medical graduates. Med. J. Aust..

[18] Backović D.V., Ilić Živojinović J., Maksimović J., Maksimović M. (2012). Gender differences in academic stress and burnout among medical students in final years of education. Psychiatr. Danub..

[19] Frajerman A., Morvan Y., Krebs M.-O., Gorwood P., Chaumette B. (2019). Burnout in medical students before residency: A systematic review and meta-analysis. Eur. Psychiatry.

[20] Liu X., Oda S., Peng X., Asai K. (1997). Life events and anxiety in Chinese medical students. Soc. Psychiatry Psychiatr. Epidemiol..

[21] Kansoun Z., Boyer L., Hodgkinson M., Villes V., Lançon C., Fond G. (2019). Burnout in French physicians: A systematic review and meta-analysis. J. Affect. Disord..

[22] Dyrbye L.N., West C.P., Satele D., Boone S., Tan L., Sloan J., Shanafelt T.D. (2014). Burnout among US medical students, residents and early career physicians relative to the general US population. Acad. Med..

[23] Ishak W., Nikravesh R., Lederer S., Perry R., Ogunyemi D., Bernstein C. (2013). Burnout in medical students: A systematic review. Clin. Teach..

[24] Lindeman B., Petrusa E., McKinley S., Hashimoto D.A., Gee D., Smink D.S., Mullen J.T., Phitayakorn R. (2017). Association of burnout with emotional intelligence and personality in surgical residents: Can we predict who is most at risk?. J. Surg. Educ..

[25] Swami M., Mathur D., Pushp B. (2013). Emotional intelligence, perceived stress and burnout among resident doctors: An assessment of the relationship. Natl. Med. J. India.

[26] Folkman S., Lazarus R.S. (1980). An analysis of coping in a middle-aged community sample. J. Health Soc. Behav..

[27] Dugani S., Afari H., Hirschhorn L.R., Ratcliffe H., Veillard J., Martin G., Lagomarsino G., Basu L., Bitton A. (2018). Prevalence and factors associated with burnout among frontline primary health care providers in low-and middle-income countries: A systematic review. Gates Open Res..

[28] Monrouxe L.V., Bullock A., Tseng H.-M., Wells S.E. (2017). Association of professional identity, gender, team understanding, anxiety and workplace learning alignment with burnout in junior doctors: A longitudinal cohort study. BMJ Open.

[29] Hankins M. (2008). The reliability of the twelve-item general health questionnaire (GHQ-12) under realistic assumptions. BMC Public Health.

[30] Goldberg D.P., Williams P. (1988). User’s Guide to the General Health Questionnaire.

[31] Shelton N.J., Herrick K.G. (2009). Comparison of scoring methods and thresholds of the general health questionnaire-12 with the edinburgh postnatal depression scale in english women. Public Health.

[32] Kristensen T.S., Borritz M., Villadsen E., Christensen K.B. (2005). The copenhagen burnout inventory: A new tool for the assessment of burnout. Work Stress.

[33] Winwood P., Winefield A. (2004). Comparing two measures of burnout among dentists in Australia. Int. J. Stress Manag..

[34] Thrush C.R., Guise J.B., Gathright M.M., Messias E., Flynn V., Belknap T., Thapa P.B., Williams D.K., Nada E.M., Clardy J.A. (2019). A one-year institutional view of resident physician burnout. Acad. Psychiatry.

[35] Chin A.R.W., Chua Y.Y., Chu M.N., Mahadi N.F., Wong M.S., Yusoff M.S.B., Lee Y.Y. (2018). Investigating validity evidence of the Malay translation of the Copenhagen burnout inventory. J. Taibah Univ. Med. Sci..

[36] Carver C.S. (1997). You want to measure coping but your protocol’ too long: Consider the brief cope. Int. J. Behav. Med..

[37] Meyer B. (2001). Coping with severe mental illness: Relations of the brief COPE with symptoms, functioning and well-being. J. Psychopathol. Behav. Assess..

[38] Ranganathan P., Pramesh C.S., Aggarwal R. (2017). Common pitfalls in statistical analysis: Measures of agreement. Perspect. Clin. Res..

[39] Mandrekar J.N. (2010). Receiver operating characteristic curve in diagnostic test assessment. J. Thorac. Oncol. Off. Publ. Int. Assoc. Study Lung Cancer.

[40] World Health Organization (2014). Human Resources for Health Country Profiles: Malaysia.

[41] Puthran R., Zhang M.W., Tam W.W., Ho R.C. (2016). Prevalence of depression amongst medical students: A meta-analysis. Med. Educ..

[42] Yusoff M.S.B., Yee L., Wei L., Meng L., Bin L., Siong T., Fuad A. (2011). A study on stress, stressors and coping strategies among Malaysian medical students. Int. J. Stud. Res..

[43] Silva S.A., Silva S.U., Ronca D.B., Gonçalves V.S.S., Dutra E.S., Carvalho K.M.B. (2020). Common mental disorders prevalence in adolescents: A systematic review and meta-analyses. PLoS ONE.

[44] Erschens R., Keifenheim K.E., Herrmann-Werner A., Loda T., Schwille-Kiuntke J., Bugaj T.J., Nikendei C., Huhn D., Zipfel S., Junne F. (2019). Professional burnout among medical students: Systematic literature review and meta-analysis. Med. Teach..

[45] Wing T., Pey Y.C., Subramaniam V., Raof A.N.A., Ting O.W., Ahmad M.H.H. (2018). Prevalence of burnout in medical and non-medical undergraduate Malaysian students in various international universities-A cross-sectional study. J. Adv. Med. Med. Res..

[46] Chin R.W.A., Chua Y.Y., Chu M.N., Mahadi N.F., Yusoff M.S.B., Wong M.S., Lee Y.Y. (2016). Prevalence of burnout among universiti sains Malaysia medical students. Educ. Med. J..

[47] Galán F., Sanmartín A., Polo J., Giner L. (2011). Burnout risk in medical students in Spain using the Maslach burnout inventory-student survey. Int. Arch. Occup. Environ. Health.

[48] Purvanova R.K., Muros J.P. (2010). Gender differences in burnout: A meta-analysis. J. Vocat. Behav..

[49] Cecil J., McHale C., Hart J., Laidlaw A. (2014). Behaviour and burnout in medical students. Med. Educ. Online.

[50] Madigan D.J., Curran T. (2021). Does burnout affect academic achievement? A meta-analysis of over 100,000 students. Educ. Psychol. Rev..

[51] Freudenberger H.J. (1976). The Staff Burn-Out Syndrome.

[52] Sharma A., Sharp D., Walker L., Monson J. (2008). Stress and burnout in colorectal and vascular surgical consultants working in the UK national health service. Psycho Oncol. J. Psychol. Soc. Behav. Dimens. Cancer.

[53] Pejušković B., Lečić-Toševski D., Priebe S., Tošković O. (2011). Burnout syndrome among physicians–The role of personality dimensions and coping strategies. Psychiatr. Danub..

[54] Ro K.E.I., Tyssen R., Hoffart A., Sexton H., Aasland O.G., Gude T. (2010). A three-year cohort study of the relationships between coping, job stress and burnout after a counselling intervention for help-seeking physicians. BMC Public Health.

[55] Rowe M.M. (2006). Four-year longitudinal study of behavioral changes in coping with stress. Am. J. Health Behav..

[56] Lemaire J.B., Wallace J.E. (2010). Not all coping strategies are created equal: A mixed methods study exploring physicians’ self reported coping strategies. BMC Health Serv. Res..

[57] Hobfoll S.E. (1989). Conservation of resources: A new attempt at conceptualizing stress. Am. Psychol..

[58] Schaufeli W.B., Maassen G.H., Bakker A.B., Sixma H.J. (2011). Stability and change in burnout: A 10-year follow-up study among primary care physicians. J. Occup. Organ. Psychol..

